# Supplementary material for: Optimized methods for scRNA-seq and snRNA-seq of skeletal muscle stored in nucleic acid stabilizing preservative
Source: Commun Biol. 2025 Jan 4;8:10. doi: 10.1038/s42003-024-07445-2 (PMC11700216; doi:10.1038/s42003-024-07445-2)
Supplement: Supplementary file 2 — Supplementary Information [file 42003_2024_7445_MOESM2_ESM.pdf]

Supplementary Figure 1.

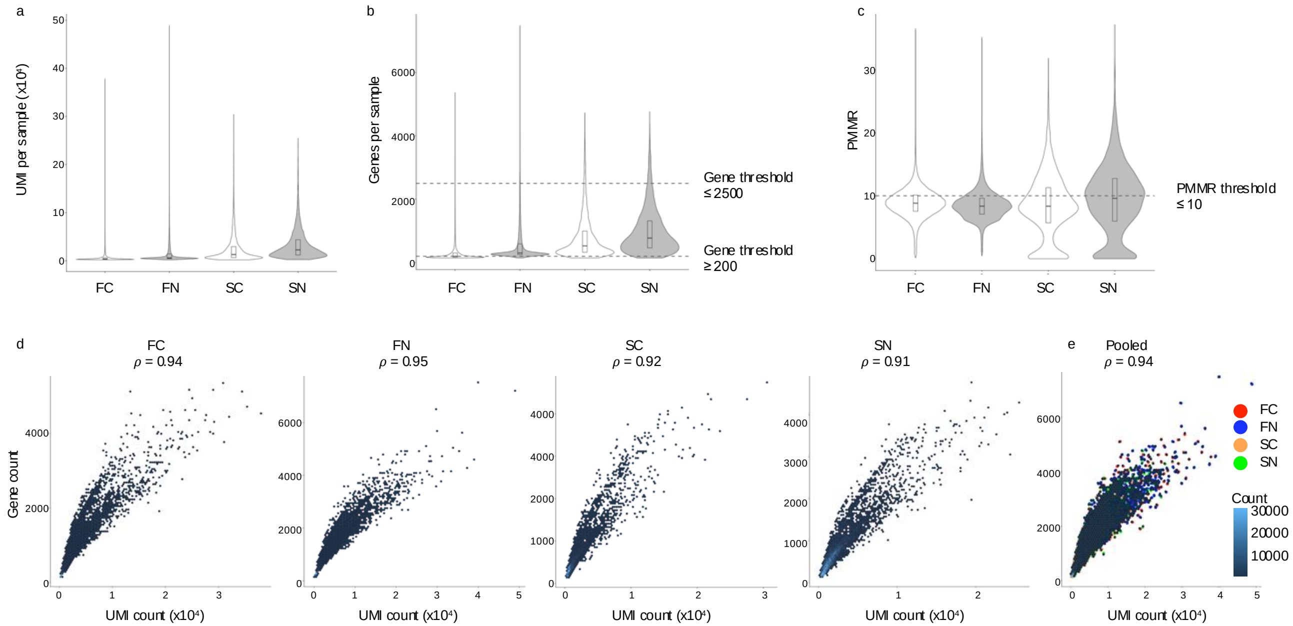

Supplementary Figure 1. Quality metrics of protocol data before filtering. a, Violin plot indicating the number of UMI detected per sample. b, Violin plot indicating the number of genes detected per sample. c, Violin plot indicating the percentage of reads mapping to mitochondrial RNAs in each sample. d, Scatter plots showing data complexity in gene count vs UMI count plots for each protocol. e, Scatter plots showing data complexity in gene count vs UMI count plots for pooled protocol data.

Supplementary Figure 2.

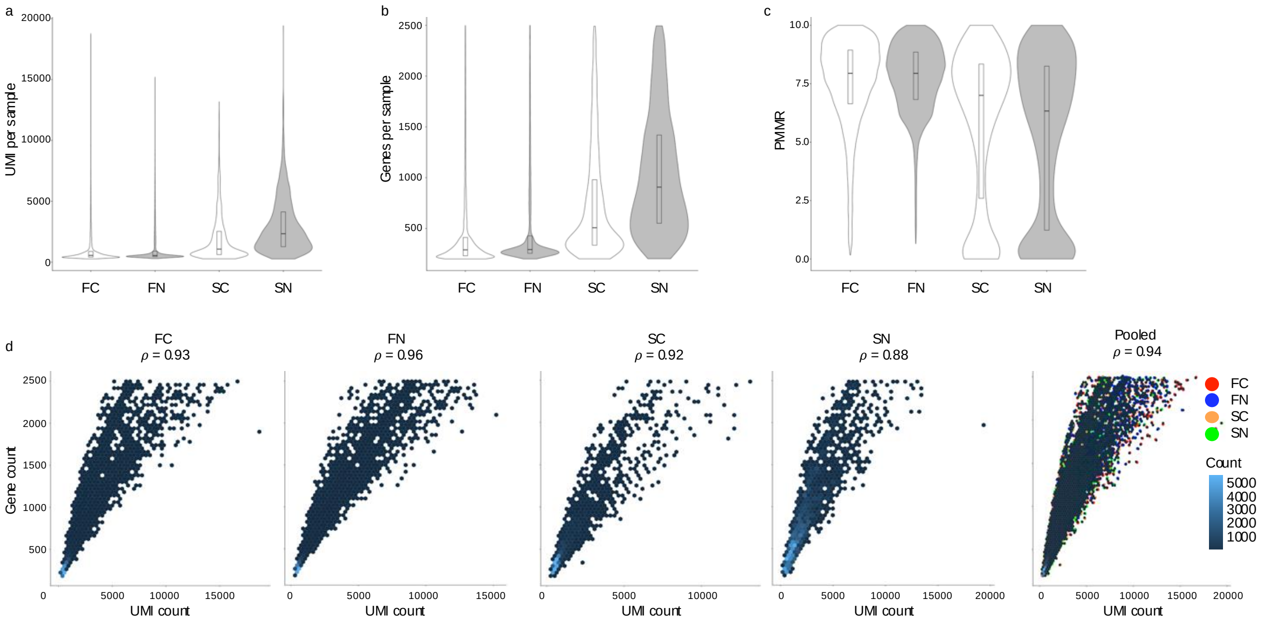

Supplementary Figure 2. Quality metrics of protocol data after filtering. a, Violin plot indicating the number of UMI detected per sample. b, Violin plot indicating the number of genes detected per sample. c, Violin plot indicating the percentage of reads mapping to mitochondrial RNAs in each sample. d, Scatter plots showing data complexity in gene count vs UMI count plots for each protocol. e, Scatter plots showing data complexity in gene count vs UMI count plots for pooled protocol data.

Supplementary Figure 3.

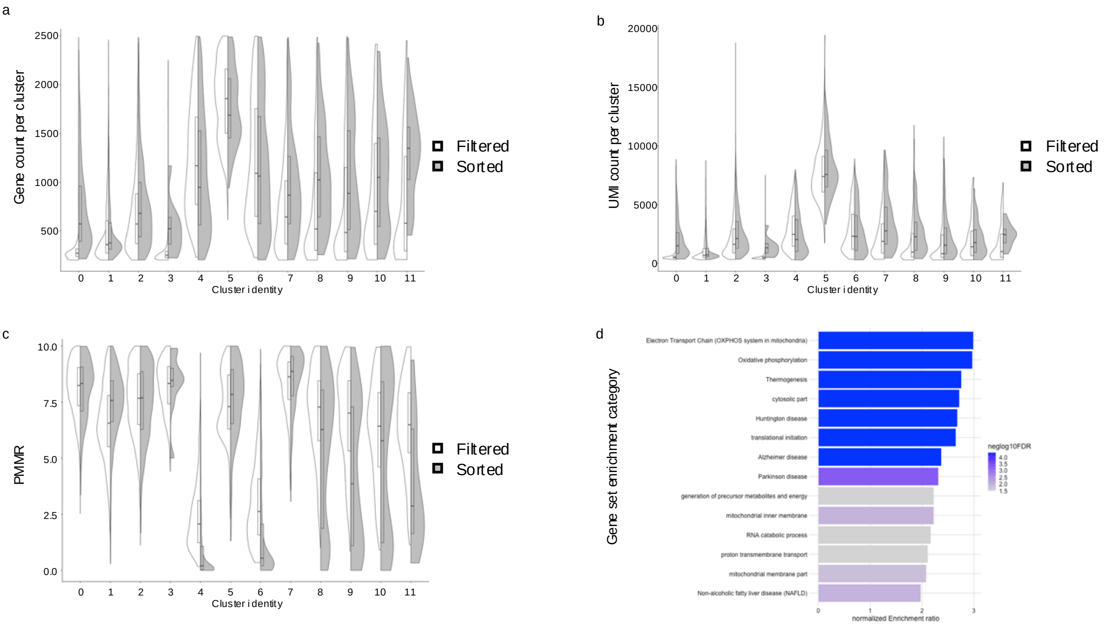

Supplementary Figure 3. Characterization of clusters 0 and 3. a, Split violin plots showing the number of genes detected per sample in either filtered (white) or sorted (gray) preparations within each cluster. b, Split violin plots showing the number of UMI detected per sample in either filtered (white) or sorted (gray) preparations within each cluster. c, Split violin plots showing the percentage of reads mapping to mitochondrial RNAs in either filtered (white) or sorted (gray) preparations within each cluster. d, Over representation analysis. *Box plots show median, 25th, and 75th percentiles.

Supplementary Figure 4.

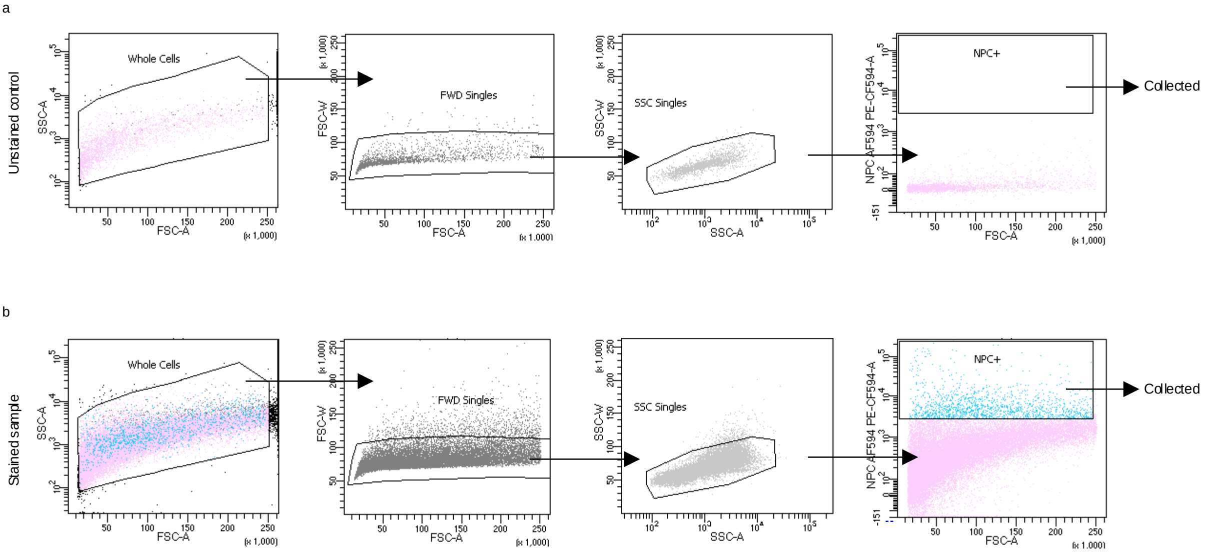

Supplementary Figure 4. Example sort logic for flow cytometric enrichment for filtered cell capture. a, Unstained control sort logic shown for whole cells after filtration. Arrows indicate gated parent cell population displayed in subsequent window. b, Stained sample sort logic shown for whole cells after filtration. Cells were collected from NPC+ gate for downstream analysis. Sort logic is identical to that used for filtered nuclei.

Supplemental Note 1: Detailed protocol of cell isolation and imaging

Reagents

Item	Supplier	Part number
AllProtect® Tissue Reagent	Qiagen	76405
Phosphate Buffered Saline (PBS) pH 7.2	Quality Biological	111-056
Bovine Serum Albumin	Millipore Sigma	A7888-50G
RNaseOUT™ Recombinant Ribonuclease Inhibitor	ThermoFisher	10777019
EASYstrainer, 70 μM, small diameter	Greiner Bio-one	542170
EASYstrainer, 40 μM, small diameter	Greiner Bio-one	542140
Nuclei Extraction Buffer	Miltenyi	130-128-024
Nuclei Buffer (20X)	10X Genomics	2000153
Alexa Fluor® 594 anti-Nuclear Pore Complex Proteins Antibody	BioLegend	682202
DAPI solution (1 mg/mL)	ThermoFisher	62248

Buffer annotation

Notation	Contents
pBSA	0.04% Bovine Serum Albumin in Phosphate Buffered Saline (pH 7.2) + 1 U/μL RNaseOUT
NucB	1X Nuclei Buffer (diluted in nuclease-free water) + 1 U/μL RNaseOUT
NEB	1X Nuclei Extraction Buffer
NPC-AF594	Alexa Fluor® 594 anti-Nuclear Pore Complex Proteins Antibody

**Buffers and solutions should be prepared and filtered through a 0.22 μm PES strainer immediately before use*
**Keep all buffers and solutions on ice*

Tissue homogenization

1. Thaw tissue stored in AllProtect® Tissue Reagent on ice
2. Serially dilute and exchange AllProtect® Tissue Reagent with PBS until all storage reagent has been removed
3. Resuspend in 0.5 mL pBSA
4. Pulverize tissue to single cell suspension with single-use microtube homogenizer
5. Filter through 70 μm strainer into fresh 1.5 mL tube
6. Rinse strainer with 0.5 mL pBSA
7. Centrifuge for 5 min at 300xg, 4°C
8. Remove supernatant and resuspend in 0.5 mL pBSA
9. Filter through 40 μm strainer into fresh 1.5 mL tube
10. Rinse strainer with 0.5 mL pBSA
11. This preparation will subsequently be referred to as filtered cells

Filtered cell (FC) capture preparation:

1. Start with filtered cells
2. Remove ~60,000 cells to fresh 1.5 mL tube
3. Centrifuge for 5 min at 300 xg, 4°C
4. Resuspend 60,000 cells in 30 µL pBSA
5. Proceed to Chromium Single Cell 5’ Library & Gel Bead Kit v2 sample capture

Filtered nuclei (FN) capture preparation:

1. Start with filtered cells
2. Remove 250,000 cells to fresh 1.5 mL tube
3. Centrifuge for 5 min at 300 xg, 4°C
4. Resuspend in 100 µL Nuclei Extraction Buffer
5. Incubate on ice, 5 min
6. Centrifuge for 5 min at 300 xg, 4°C
7. Resuspend in 30 µL Nuclei Buffer
8. Proceed to Chromium Single Cell 5’ Library & Gel Bead Kit v2 sample capture

Staining and flow cytometry enrichment

1. Start with filtered cells
2. Remove 50,000 cells as an unstained cell control (bring minimum volume to 100 µL)
3. Stain 500,000 cells with a final concentration of 25 µg/mL NPC-AF594
4. Incubate on ice, 30 min
5. Bring all volumes to 1 mL
6. Centrifuge for 5 min at 300xg, 4°C
7. Resuspend in 300 µL pBSA
8. Sort NPC-AF594+ cells on a Becton Dickinson FACS Aria Fusion using 70 µm nozzle into a collection tube on ice
9. This preparation will subsequently be referred to as sorted cells

Sorted cell (SC) capture preparation:

1. Start with sorted cells
2. Remove ~100,000 cells to fresh 1.5 mL tube
3. Centrifuge for 5 min at 300 xg, 4°C
4. Resuspend 60,000 cells in 30 µL pBSA
5. Proceed to Chromium Single Cell 5’ Library & Gel Bead Kit v2 sample capture

Sorted nuclei (SN) capture preparation:

1. Start with sorted cells
2. Remove 250,000 cells to fresh 1.5 mL tube
3. Centrifuge for 5 min at 300 xg, 4°C
4. Resuspend in 100 µL Nuclei Extraction Buffer

5. Incubate on ice, 5 min
6. Centrifuge for 5 min at 300 xg, 4°C
7. Resuspend in 30 µL Nuclei Buffer
8. Proceed to Chromium Single Cell 5' Library & Gel Bead Kit v2 sample capture

Staining for confocal microscopy

1. Start with up to 50,000 sorted cells (sorted cell capture preparation) or 50,000 sorted nuclei (sorted nuclei capture preparation) in 100 µL pBSA
2. Stain with a final concentration of 100 µg/mL NPC-AF594 + 50 µg/mL DAPI
3. Incubate on ice, 20 min
4. Centrifuge for 5 min at 300 xg, 4°C
5. Resuspend in 300 µL pBSA
6. Transfer suspension to a 35 mm 1.5 coverslip dish
7. Imaging was performed on an LSM 880 Airyscan Confocal microscope (Zeiss) with a 63x/1.4 oil objective and captured with Zen_2.3 software (Zeiss).
