# Supplementary material for: Optimized methods for scRNA-seq and snRNA-seq of skeletal muscle stored in nucleic acid stabilizing preservative
Source: Commun Biol. 2025 Jan 4;8:10. doi: 10.1038/s42003-024-07445-2 (PMC11700216; doi:10.1038/s42003-024-07445-2)
Supplement: Supplementary file 3 — Description of Additional Supplementary Files [file 42003_2024_7445_MOESM3_ESM.pdf]

Description of Additional Supplementary Files

File name: Supplementary Data 1-4
Description: Source data behind the graphs in the paper
