# Supplementary material for: Optimized methods for scRNA-seq and snRNA-seq of skeletal muscle stored in nucleic acid stabilizing preservative
Source: Commun Biol. 2025 Jan 4;8:10. doi: 10.1038/s42003-024-07445-2 (PMC11700216; doi:10.1038/s42003-024-07445-2)
Supplement: Supplementary file 5 — Reporting summary [file 42003_2024_7445_MOESM5_ESM.pdf]

nature portfolio

Corresponding author(s): DBPR COMMSBIO-24-3889-T

Last updated by author(s): Jul 22, 2024

Reporting Summary

Nature Portfolio wishes to improve the reproducibility of the work that we publish. This form provides structure for consistency and transparency in reporting. For further information on Nature Portfolio policies, see our Editorial Policies and the Editorial Policy Checklist.

Statistics

For all statistical analyses, confirm that the following items are present in the figure legend, table legend, main text, or Methods section.

n/a	Confirmed	
☐	☒	The exact sample size (*n*) for each experimental group/condition, given as a discrete number and unit of measurement
☐	☒	A statement on whether measurements were taken from distinct samples or whether the same sample was measured repeatedly
☐	☒	The statistical test(s) used AND whether they are one- or two-sided<br>*Only common tests should be described solely by name; describe more complex techniques in the Methods section.*
☒	☐	A description of all covariates tested
☐	☒	A description of any assumptions or corrections, such as tests of normality and adjustment for multiple comparisons
☐	☒	A full description of the statistical parameters including central tendency (e.g. means) or other basic estimates (e.g. regression coefficient) AND variation (e.g. standard deviation) or associated estimates of uncertainty (e.g. confidence intervals)
☐	☒	For null hypothesis testing, the test statistic (e.g. F, t, r) with confidence intervals, effect sizes, degrees of freedom and P value noted<br>*Give P values as exact values whenever suitable.*
☒	☐	For Bayesian analysis, information on the choice of priors and Markov chain Monte Carlo settings
☒	☐	For hierarchical and complex designs, identification of the appropriate level for tests and full reporting of outcomes
☐	☒	Estimates of effect sizes (e.g. Cohen's *d*, Pearson's *r*), indicating how they were calculated

Our web collection on statistics for biologists contains articles on many of the points above.

Software and code

Policy information about availability of computer code

Data collection: No software was used to collect data

Data analysis: FASTQ files were demultiplexed in Cell Ranger_7.2.0 (10x Genomics) and mapped to the GRCh38-2020-A transcriptome. Ambient RNA excluded with CellBender_0.3.0 22. Quality control and clustering was performed with Seurat_5.0.1 23, SeuratObject_5.0.0, and DoubletFinder_2.0.3 24. Filtering was performed as follows: nFeature_RNA ≥ 200, nFeature_RNA ≤ 2500, percentage reads mapped mitochondrial RNAs (PMMR) ≤ 10. Predicted doublet rate was set at 0.05. Because all specimens were derived from the same starting material and sequenced in a single batch, data were log normalized with a scale factor of 10000. Differential abundance testing was performed with MiloR_1.8.1 18. Over representation analyses (ORA) and gene set enrichment analyses (GSEA) were performed in WebGestalt_0.4.6 25. Additional R software packages are listed below:

R version 4.3.2 (2023-10-31)
Platform: aarch64-apple-darwin20 (64-bit)
Running under: macOS Ventura 13.6.4

attached base packages:
[1] stats graphics grDevices utils datasets methods base

other attached packages:
[1] DoubletFinder_2.0.4 ggplot2_3.4.4 cowplot_1.1.3 patchwork_1.2.0 dplyr_1.1.4 Seurat_5.0.1 SeuratObject_5.0.1
[8] sp_2.1-3

```
loaded via a namespace (and not attached):
 [1] deldir_2.0-2        pbapply_1.7-2        gridExtra_2.3        rlang_1.1.3         magrittr_2.0.3       RcppAnnoy_0.0.22
 [7] spatstat.geom_3.2-8   matrixStats_1.2.0    ggridges_0.5.6       compiler_4.3.2       png_0.1-8            vctrs_0.6.5
[13] reshape2_1.4.4      stringr_1.5.1        pkgconfig_2.0.3      shape_1.4.6          fastmap_1.1.1        ellipsis_0.3.2
[19] utf8_1.2.4          promises_1.2.1       purrr_1.0.2          jsonlite_1.8.8       goftest_1.2-3        later_1.3.2
[25] spatstat.utils_3.0-4  irlba_2.3.5.1      parallel_4.3.2       cluster_2.1.6        R6_2.5.1             ica_1.0-3
[31] stringi_1.8.3       RColorBrewer_1.1-3   spatstat.data_3.0-4  reticulate_1.35.0    parallelly_1.36.0    lmtest_0.9-40
[37] scattermore_1.2     Rcpp_1.0.12          tensor_1.5           future.apply_1.11.1  zoo_1.8-12           sctransform_0.4.1
[43] httpuv_1.6.14       Matrix_1.6-5         splines_4.3.2        igraph_2.0.1.1       tidyselect_1.2.0     abind_1.4-5
[49] rstudioapi_0.15.0     spatstat.random_3.2-2  codetools_0.2-19   miniUI_0.1.1.1       spatstat.explore_3.2-6 listenv_0.9.1
[55] lattice_0.22-5      tibble_3.2.1         plyr_1.8.9           withr_3.0.0          shiny_1.8.0          ROCR_1.0-11
[61] Rtsne_0.17          future_1.33.1        fastDummies_1.7.3    survival_3.5-7       polyclip_1.10-6      zip_2.3.1
[67] fitdistrplus_1.1-11  circlize_0.4.15     pillar_1.9.0         KernSmooth_2.23-22   plotly_4.10.4        generics_0.1.3
[73] RcppHNSW_0.6.0      munsell_0.5.0        scales_1.3.0         globals_0.16.2       xtable_1.8-4         glue_1.7.0
[79] lazyeval_0.2.2      tools_4.3.2          data.table_1.15.0    RSpectra_0.16-1      openxlsx_4.2.5.2     RANN_2.6.1
[85] leiden_0.4.3.1      dotCall64_1.1-1      grid_4.3.2           tidyr_1.3.1          colorspace_2.1-0     nlme_3.1-164
[91] cli_3.6.2           spatstat.sparse_3.0-3  spam_2.10-0        fansi_1.0.6          viridisLite_0.4.2    uwot_0.1.16
[97] gtable_0.3.4        digest_0.6.34        progressr_0.14.0     ggrepel_0.9.5        htmlwidgets_1.6.4    htmltools_0.5.7
[103] lifecycle_1.0.4    httr_1.4.7           GlobalOptions_0.1.2  mime_0.12            MASS_7.3-60.0.1
```

For manuscripts utilizing custom algorithms or software that are central to the research but not yet described in published literature, software must be made available to editors and reviewers. We strongly encourage code deposition in a community repository (e.g. GitHub). See the Nature Portfolio guidelines for submitting code & software for further information.

Data

Policy information about availability of data

All manuscripts must include a data availability statement. This statement should provide the following information, where applicable:

- Accession codes, unique identifiers, or web links for publicly available datasets
- A description of any restrictions on data availability
- For clinical datasets or third party data, please ensure that the statement adheres to our policy

Accession codes will be made available before publication

Human research participants

Policy information about studies involving human research participants and Sex and Gender in Research.

Reporting on sex and gender	Sex and gender were not reported in this study
Population characteristics	De-identified biopsied skeletal muscle tissue from patients without type 2 diabetes were used.
Recruitment	Ethical approval for the AADM study under which the samples were collected was obtained from the National Institutes of Health (09-HG-N070) and the National Health Research Ethics Committee of Nigeria (NHREC). Informed consent was obtained from all participants prior to enrollment. The research adhered to the Declaration of Helsinki
Ethics oversight	National Institutes of Health (09-HG-N070); National Health Research Ethics Committee of Nigeria (NHREC)

Note that full information on the approval of the study protocol must also be provided in the manuscript.

Field-specific reporting

Please select the one below that is the best fit for your research. If you are not sure, read the appropriate sections before making your selection.

☒ Life sciences ☐ Behavioural & social sciences ☐ Ecological, evolutionary & environmental sciences

For a reference copy of the document with all sections, see nature.com/documents/nr-reporting-summary-flat.pdf

Life sciences study design

All studies must disclose on these points even when the disclosure is negative.

Sample size	No sample-size calculations were performed. Three samples were chosen at random from a de-identified list, tissue was pooled, and a subset of the pool equaling approximately 100mg was extracted for further experimentation.
Data exclusions	No data were excluded from the analysis.

Replication	Experimental findings were confirmed via four data processing pipelines.
Randomization	No randomized studies were performed.
Blinding	No blinding was used since experiments were performed on de-identified biopsied tissue.

Reporting for specific materials, systems and methods

We require information from authors about some types of materials, experimental systems and methods used in many studies. Here, indicate whether each material, system or method listed is relevant to your study. If you are not sure if a list item applies to your research, read the appropriate section before selecting a response.

Materials & experimental systems

n/a	Involved in the study
☐	☒ Antibodies
☒	☐ Eukaryotic cell lines
☒	☐ Palaeontology and archaeology
☒	☐ Animals and other organisms
☒	☐ Clinical data
☒	☐ Dual use research of concern

Methods

n/a	Involved in the study
☒	☐ ChIP-seq
☐	☒ Flow cytometry
☒	☐ MRI-based neuroimaging

Antibodies

Antibodies used	Alexa Fluor® 594 (NPC-AF594) antibody (BioLegend, San Diego, CA, catalogue no. 682202)
Validation	All primary conjugated antibodies were validated for specificity by the suppliers. Validation methods are supplied on the manufacturer websites.

Flow Cytometry

Plots

Confirm that:

☒ The axis labels state the marker and fluorochrome used (e.g. CD4-FITC).

☒ The axis scales are clearly visible. Include numbers along axes only for bottom left plot of group (a 'group' is an analysis of identical markers).

☒ All plots are contour plots with outliers or pseudocolor plots.

☒ A numerical value for number of cells or percentage (with statistics) is provided.

Methodology

Sample preparation	Tissue was obtained from a single percutaneous needle biopsy of the vastus lateralis muscle. Tissue was immediately dissected into skeletal muscle and adipose tissue parts and placed separately in Allprotect® Tissue Reagent (ATR) (Qiagen, Germantown, MD, catalogue no. 76405) to stabilize tissue RNA, DNA and proteins. Tissues were transitioned to -80□C until shipped on dry ice from the study site (Ibadan, Nigeria) to the Center for Research on Genomics and Global Health at the National Institutes of Health (Bethesda, MD, USA). Skeletal muscle specimens stored in Allprotect® Tissue Reagent (ATR) were thawed on ice. Ice cold PBS was added and pipette-mixed with ATR to dilute and remove supernatant. This process was repeated until all ATR was removed. Tissue was subsequently rinsed twice in ice cold PBS to remove any remaining ATR, weighed, and resuspended in pBSA (PBS + 0.04% BSA) + 1U/mL RNaseOut (RNaseOUT™ Recombinant RNase Inhibitor, Thermo Fisher Scientific, Waltham, MA, catalogue no. 10777019). Manual tissue dissociation was performed with a disposable micro-tissue homogenizer. Cell concentrations were estimated with a hemocytometer. Single cell suspensions were strained sequentially through 70 µm and 40 µm filters, with washes between each step (“filtration alone” samples).<br>For sorted samples, cells were stained with a final concentration of 25 µg/mL anti-Nuclear Pore Complex conjugated to Alexa Fluor® 594 (NPC-AF594) antibody (BioLegend, San Diego, CA, catalogue no. 682202) at 4℃, 30 minutes. Excess antibody was removed and NPC+ cells isolated on a Becton Dickinson FACS ARIA Fusion using a 70 µm nozzle.<br>For nuclei isolation, cells were incubated with Nuclear Extraction Buffer (Miltenyi Biotec, Gaithersburg, MD, catalogue no. 130-128-024) containing 1 U/mL RNaseOut at 4℃ for 5 minutes. Following the incubation, suspensions were centrifuged at 300 x g, 4°C, 5 minutes, supernatant removed, and nuclei resuspended in pBSA. Samples were sorted on ice into pBSA before downstream processing. Note that “sorted nuclei” refers to cells that were sorted prior to nuclei extraction.
Instrument	Becton Dickinson FACS ARIA Fusion using a 70 µm nozzle

Software	BD FACS DIVA software
Cell population abundance	Post-sort purity was determined by re-analyzing sorted cells and visually by confocal microscopy. Post-sort analysis demonstrated 85% - 90% purity.
Gating strategy	Cells were gated on FSC/SSC, followed by FSC-A/FSC-W to SSC-A/SSC-W. Sorted cells were collected by NPC-AF594/FSC-A positivity, where positivity was determined against unstained sample processed in parallel.

☒ Tick this box to confirm that a figure exemplifying the gating strategy is provided in the Supplementary Information.
